# Supplementary material for: Gut acquisition of Extended-spectrum β-lactamases-producing Klebsiella pneumoniae in preterm neonates: Critical role of enteral feeding, and endotracheal tubes in the neonatal intensive care unit (NICU)
Source: PLoS One. 2023 Nov 7;18(11):e0293949. doi: 10.1371/journal.pone.0293949 (PMC10629629; doi:10.1371/journal.pone.0293949)
Supplement: Attachment — Submitted filename: rebuttal letter..docx [file pone.0293949.s002.docx]

Moussa Benboubker

Human Pathology Biomedicine and Environment Laboratory

Faculty of Medicine and Pharmacy

Sidi Mohammed Ben Abdellah University, Fez, Morocco

**Rebuttal Letter**

Dear Editor,

We were pleased to have an opportunity to revise our manuscript now entitled “Gut acquisition of Extended-spectrum β-lactamases-producing Klebsiella pneumoniae in preterm neonates: critical role of enteral feeding, and endotracheal tubes in the neonatal intensive care unit (NICU)” (PONE-D-22-25668). In the revised manuscript, we have carefully considered the reviewers’ comments and suggestions. As instructed, we have attempted to succinctly explain the changes made in response to all the comments.

We have added the revised manuscript as recommended. Responses to the concerns raised by the reviewers are presented below in the table and in the revised text with marked-up copy.

The reviewers’ comments were very helpful overall, and we are appreciative of such constructive feedback on our original submission. After addressing the issues raised, we feel the quality of the paper is much improved.

*Sincerely*

*Moussa Benboubker*

| **Points raised by reviewer(s)** | **Responses** |
| --- | --- |
| Lines 32, 256: “K. Pneumoniae” should be written in italic | "K. Pneumoniae" has been converted to italics |
| Line 54: There is unnecessary dot after the word “Klebsiella” | Dot has been deleted |
| Reference list has a some gaps such us year of article in point 4 and 7. It should list should be checked carefully. | Both existing references are not correctly displayed on the list with the style recommended by the journal automaticully, so we have replaced references 4 and 7 with other references that begin in the same context in the manuscript and are presented as follows:  **Reference 4** [Cotton MF, Wasserman E, Pieper CH, Theron DC, van Tubbergh D, Campbell G, et al. Invasive disease due to extended spectrum beta-lactamase-producing Klebsiella pneumoniae in a neonatal unit:the possible role of cockroaches. :5.] has been replaced by [Pavez M, Troncoso C, Osses I, Salazar R, Illesca V, Reydet P, et al. High prevalence of CTX-M-1 group in ESBL-producing enterobacteriaceae infection in intensive care units in southern Chile. Braz J Infect Dis. 2019 Apr 24;23(2):102–10.]  **And reference 7** [Sakai AM, Iensue TNAN, Pereira KO, Silva RL da, Pegoraro LG de O, Salvador MS de A, et al. Colonization profile and duration by multi-resistant organisms in a prospective cohort of newborns after hospital discharge. Rev Inst Med Trop São Paulo. 2020;62:e22.] has been remplaced by[Milic M, Siljic M, Cirkovic V, Jovicevic M, Perovic V, Markovic M, et al. Colonization with Multidrug-Resistant Bacteria in the First Week of Life among Hospitalized Preterm Neonates in Serbia: Risk Factors and Outcomes. Microorganisms. 2021 Dec 17;9(12):2613.] |
